# Helminth Infection, Gut Microbiome Alterations, and Their Impact on Pulmonary Tuberculosis Susceptibility

**DOI:** 10.1155/jotm/3767562

**Published:** 2026-04-29

**Authors:** Afiat Berbudi, Silvita Fitri Riswari, Alexander Kwarteng

**Affiliations:** ^1^ Department of Biomedical Sciences, Faculty of Medicine, Universitas Padjadjaran, Bandung, Indonesia, unpad.ac.id; ^2^ Research Center for Care and Control of Infectious Diseases (RC3ID), Universitas Padjadjaran, Bandung, Indonesia, unpad.ac.id; ^3^ Department of Biochemistry and Biotechnology, Kwame Nkrumah University of Science and Technology, Kumasi, Ghana, knust.edu.gh

**Keywords:** gut–lung axis, helminths, immune modulation, microbiome, pulmonary tuberculosis, SCFAs

## Abstract

**Background:**

Helminth infections and pulmonary tuberculosis (TB) frequently coexist in low‐ and middle‐income countries and interact through immune‐mediated mechanisms that influence host susceptibility to *Mycobacterium tuberculosis* (*Mtb*). Beyond direct immunomodulation, increasing evidence indicates that helminth infections alter gut microbiome composition and microbial metabolite production, thereby shaping systemic and pulmonary immune responses through the gut–lung axis. Given the central role of the gut microbiome in regulating T‐cell polarization, macrophage function, and inflammatory balance, microbiome‐mediated pathways have emerged as a potential link between helminth infection and impaired host defense against pulmonary TB.

**Objectives:**

This narrative review examines current evidence on how helminth‐induced immunological changes and gut microbiome alterations, within the context of the gut–lung axis, may influence susceptibility to pulmonary TB.

**Methods:**

A narrative review approach was used to synthesize findings from experimental, observational, and clinical studies addressing helminth infection, gut microbiome dynamics, immune regulation, and TB.

**Results:**

Helminth infections are associated with Th2‐skewed immune responses characterized by increased regulatory T‐cell activity and anti‐inflammatory cytokine production, which may attenuate Th1‐mediated immunity essential for *Mtb* control. Helminths also modulate gut microbiome composition, with effects ranging from increased microbial diversity to dysbiosis, depending on helminth species and host context. These microbiome alterations may influence systemic immunity through microbial metabolites such as short‐chain fatty acids (SCFAs). Importantly, SCFAs exhibit context‐dependent effects, potentially supporting immune homeostasis while, under certain conditions, promoting regulatory pathways that may dampen protective antimycobacterial responses.

**Conclusions:**

Current evidence suggests that helminth‐associated immune modulation and gut microbiome alterations may influence pulmonary TB susceptibility, although most findings remain associative rather than causal. Further mechanistic and clinical studies are needed to clarify the role of the gut–lung axis in helminth–TB coinfection and to inform integrated disease management strategies in endemic regions.

## 1. Introduction

Helminth infections and tuberculosis (TB) remain major public health burdens in many low‐ and middle‐income countries, where they frequently overlap in geographical distribution and disproportionately affect socioeconomically disadvantaged populations. Soil‐transmitted helminths such as *Ascaris lumbricoides*, hookworms, and *Trichuris trichiura* infect more than one billion people worldwide, with particularly high prevalence in Sub‐Saharan Africa and Southeast Asia [1, 2]. In parallel, TB continues to represent a major global health challenge. According to [3], an estimated 10.7 million people developed TB in 2024, with the highest burden occurring in regions where helminth infections are also highly endemic [3]. This geographical overlap increases the likelihood of helminth–TB coinfections and highlights the need to better understand how these infections interact within affected populations.

Recent evidence highlights the relevance of the gut–lung axis in shaping immune responses during helminth and TB coinfection. This axis represents a bidirectional communication network through which gut microbial communities and their metabolites influence pulmonary immunity, thereby affecting susceptibility to *Mycobacterium tuberculosis* (*Mtb*) [4–6]. Helminth infections are well known to induce a T‐helper type 2 (Th2)‐dominated immune profile characterized by IL‐4, IL‐5, and IL‐13, accompanied by increased regulatory T‐cell (Treg) activity and IL‐10/TGF‐β production [7, 8]. Such immunological shifts have been associated with attenuation of Th1‐mediated responses that are essential for *Mtb* control, potentially influencing host susceptibility to pulmonary TB.

Helminths also alter the gut microbiome, although findings remain context‐dependent across species and study designs. Some studies report increased microbial diversity and a relative enrichment of beneficial taxa during helminth infection [9], while others describe dysbiosis marked by reduced diversity and a shift toward opportunistic organisms [10]. These alterations may influence systemic immune regulation through microbial metabolites, including short‐chain fatty acids (SCFAs), which have been implicated in modulating Treg differentiation, macrophage activation, and inflammatory signaling [11, 12]. However, the extent to which helminth‐associated microbiome alterations directly influence pulmonary TB outcomes in humans remains incompletely understood.

Although several reviews have discussed helminth‐induced immunomodulation in TB [13], few have comprehensively integrated helminth‐driven immunological effects with gut microbiome dynamics and gut–lung axis mechanisms within a unified conceptual framework. Important gaps persist, including the context‐specific effects of SCFAs on TB immunity, the extent to which helminth‐mediated dysbiosis shapes pulmonary responses, and the differential roles of species‐specific helminth burdens. Addressing these gaps is critical for understanding how coinfections influence TB susceptibility and treatment responses in endemic settings.

This narrative review synthesizes current evidence on how helminth infections modulate immune pathways and alter the gut microbiome, thereby influencing susceptibility to pulmonary TB. By bridging helminth immunobiology, microbial ecology, and TB pathogenesis, this review aims to clarify emerging mechanisms, identify unresolved questions, and discuss potential implications for disease management in helminth‐endemic regions.

## 2. Helminth Infection and the Immune System

### 2.1. Immunomodulatory Effects of Helminths

Helminths significantly influence host immunity by switching the immune response towards a Th2 profile. This Th2‐dominant profile involves increased IL‐4, IL‐5, and IL‐13, which favor helminth survival but may downregulate Th1‐mediated immunity required for controlling intracellular pathogens such as *Mtb* [14]. Helminths also promote the activity of regulatory T cells (Tregs), which release immunosuppressive cytokines like IL‐10 and TGF‐β, thereby fostering immune tolerance and potentially reducing proinflammatory responses needed for pathogen containment [8, 15].

These immune shifts, while beneficial for helminth persistence, may alter host susceptibility to other pathogens including *Mtb*, although their effects can vary depending on infection intensity and host context.

Th2 responses are associated with alternatively activated macrophages (AAMs), which emphasize tissue repair and regulatory functions rather than microbicidal activity, potentially limiting the Th1‐driven antimicrobial mechanisms required for *Mtb* control [16, 17]. Helminth‐induced AAM polarization may therefore dampen macrophage killing capacity and reduce the strength of *Mtb*‐specific Th1 responses [18–20].

Helminth‐induced Tregs further suppress proinflammatory responses by increasing IL‐10 and TGF‐β, contributing to a regulatory environment that limits excessive inflammation but may concurrently impair optimal antimicrobial immunity [21]. While such tolerance supports helminth survival, it may reduce the host’s capacity to mount protective responses against secondary infections, including TB [22]. Moreover, helminths induce a Th2‐dominant environment by releasing glycoproteins that interfere with normal immune signaling pathways, thereby promoting their survival. These helminth‐derived molecules can modulate dendritic cells and macrophages, reinforcing Th2 polarization and regulatory pathways [23, 24].

### 2.2. Impact on TB Immune Response

Helminth infections compromise the host’s immune response to TB by promoting Th2 polarization, thereby modulating or dampening Th1 pathways that are essential for *Mtb* containment [25, 26]. Effective TB immunity relies on interferon‐gamma (IFN‐γ) production to activate macrophages to contain *Mtb*. Helminth infections can elevate IL‐4, IL‐5, IL‐10, and TGF‐β, which may downregulate IFN‐γ–mediated macrophage activation and reduce the efficiency of *Mtb* killing [27, 28].

Furthermore, helminth antigens may impair macrophage function, affecting phagosome maturation and hindering *Mtb* containment [25, 29]. Helminth‐driven modulation of macrophages can also reduce the effectiveness of *Mtb*‐specific CD4+ T‐cell responses, further influencing TB immunity [30]. By altering macrophage activity, helminths create a favorable environment for TB to persist, potentially leading to more severe disease manifestations [31].

Increased Tregs also contribute to immune suppression, further impairing the Th1 response by releasing IL‐10 and TGF‐β to suppress the inflammatory responses essential for TB control [32, 33]. Collectively, these effects may influence TB progression and treatment outcomes, although the magnitude of impact likely varies across helminth species, infection load, and host immune status [26, 31]. The interplay of Tregs and Th2 cytokines limits the host’s ability to mount an effective anti‐TB response, facilitating TB persistence. Consequently, these helminth‐induced immunomodulations create an environment conducive to their survival but at the cost of increased vulnerability to TB.

Helminth coinfections have been associated with altered TB severity and may affect the likelihood of latent TB reactivation in some contexts [13, 21]. However, evidence remains largely associative, and further mechanistic studies are needed to clarify causal pathways. Such interactions highlight the potential importance of integrated approaches to manage TB in helminth‐endemic settings.

### 2.3. Coinfection Prevalence and Clinical Implications

The prevalence of helminth–TB coinfections varies widely, with substantial rates reported in regions where both diseases are endemic. In Ethiopia, for example, TB patients often experience coinfection rates of up to 71%, highlighting the high disease burden [14, 32]. Similarly, studies in Tanzania have shown significant rates of helminth coinfection among TB patients, with malnutrition commonly exacerbating the severity of both infections [34, 35].

Helminth coinfections may complicate TB management by altering cytokine profiles, potentially reducing Th1 responses required for effective *Mtb* control [31]. Some studies suggest that helminth‐induced immune modulation may affect macrophage activation, systemic inflammation, or drug pharmacodynamics, although data remain limited and context‐specific [36, 37].

Co‐infected individuals may exhibit altered clinical presentations and, in some cases, more extensive disease, although the extent of helminth contribution remains under investigation [31]. Associations between helminth infection and increased risk of active TB have been reported [31, 38], but further epidemiological studies are required to quantify species‐specific and population‐level effects.

## 3. Gut Microbiome and Its Role in Immune Regulation

### 3.1. Overview of Gut Microbiome Composition

The gut microbiome is a complex ecosystem of microorganisms, predominantly comprising five major bacterial phyla: Firmicutes, Bacteroidetes, Actinobacteria, Proteobacteria, and Verrucomicrobiota (Table 1). These bacteria contribute to numerous physiological processes essential for maintaining health and regulating the immune system. The diversity and relative balance of these phyla are important for gut homeostasis, and alterations in their abundance have been associated with immune dysregulation and disease susceptibility [39–41].

**TABLE 1:** Major gut microbiome phyla and their immunoregulatory roles.

Bacteria phyla	Key immunological roles	Mechanism of action	Reference
Firmicutes	Promote anti‐inflammatory responses and gut homeostasis	Enhance Treg‐cell induction and production of butyrate, an SCFA with anti‐inflammatory properties	[40–42]
Proteobacteria	Proinflammatory signaling	LPS‐mediated innate immune activation	[44]
Bacteroidetes	Immune modulation	SCFA production, polysaccharide metabolism	[39, 42]
Actinobacteria	Gut barrier and immune development	Acetate production, IgA regulation	[39, 40]
Verrucomicrobiota	Barrier integrity	Mucin degradation, *Akkermansia*‐mediated epithelial support	[45]

Firmicutes, encompassing genera like *Lactobacillus* and *Clostridium*, play a key role in carbohydrate metabolism and SCFA production, such as butyrate, propionate, and acetate. These SCFAs not only serve as energy sources for colonic cells but also regulate intestinal pH and support anti‐inflammatory processes [40, 42]. Butyrate is particularly significant for maintaining gut barrier integrity, enhancing tight junction protein production, and preventing systemic inflammation by blocking pathogen translocation [41].

Bacteroidetes, including *Bacteroides* and *Prevotella*, also contribute to SCFA production by degrading plant polysaccharides, which are otherwise indigestible by human enzymes. Their metabolic activity promotes gut health and supports immune function by producing molecules that foster anti‐inflammatory responses [39]. These taxa are therefore implicated in immune modulation and maintenance of mucosal immune balance [40, 42].

While less abundant, Actinobacteria, including *Bifidobacterium,* contribute to immune development and vitamin production. Known for its beneficial effects, *Bifidobacterium* aids in gut barrier function, antimicrobial compound production, and modulation of gut pH [40, 43] *Actinobacteria* also produce SCFAs, particularly acetate, which may further support gut health and immune homeostasis [39].

Proteobacteria are typically present in lower abundance in a healthy gut microbiome, playing a role in nutrient processing and immune interactions. Increased Proteobacteria abundance is often associated with dysbiosis and inflammatory states, partly due to lipopolysaccharide production that can activate innate immune responses [44].

Verrucomicrobiota, though less prominent, includes *Akkermansia muciniphila*, a bacterium associated with gut barrier maintenance and metabolic health. It resides in the gut mucus layer, degrades mucin, and contributes to epithelial barrier integrity. These functions are associated with metabolic regulation and immune modulation [45].

The gut microbiome influences the development of gut‐associated lymphoid tissue (GALT) and the production of Tregs, which contribute to immune tolerance and limitation of excessive inflammation [46, 47]. Disruptions in microbiome composition (dysbiosis) may compromise these immunoregulatory functions and have been associated with an increased risk of inflammatory conditions [48, 49].

### 3.2. Gut Microbiome and Immune Regulation

The gut microbiome interacts with the immune system through multiple mechanisms, including microbial metabolites, pattern‐recognition receptor signaling, and maintenance of gut barrier integrity. SCFAs, such as butyrate, acetate, and propionate, play an important role in immune regulation by modulating inflammatory signaling pathways and T‐cell differentiation [50].

SCFAs produced by microbial fermentation of dietary fibers are key metabolites that mediate the microbiome’s effects on immunity. SCFAs such as acetate, propionate, and butyrate exert anti‐inflammatory effects by bolstering Treg function, thus supporting immune balance and preventing autoimmune reactions [51]. Furthermore, SCFAs enhance gut barrier integrity by promoting tight junction proteins, reducing the translocation of microbial products like lipopolysaccharides (LPS) into the bloodstream, and preventing systemic inflammation [44, 52].

Beyond SCFAs, the gut microbiome influences cytokine production, modulating both pro‐ and anti‐inflammatory cytokines to help control chronic inflammation associated with the metabolic syndrome and cardiovascular disorders [53, 54]. It also impacts immunoglobulin A (IgA) production, which is essential for mucosal immunity, as IgA neutralizes pathogens and promotes immune tolerance [55–60]. This interaction supports a balanced immune environment and reduces the risk of autoimmune responses.

Dysbiosis, an imbalance in the microbiome, can lead to increased gut permeability, allowing endotoxins like LPS to enter the bloodstream and trigger chronic inflammation, a hallmark of autoimmune and metabolic diseases [44, 61–65]. A healthy microbiome, however, maintains immune homeostasis, supporting lung immunity and reducing susceptibility to respiratory pathogens by preserving the integrity of the gut barrier and preventing pathogenic overgrowth [66–69].

A well‐functioning gut microbiome is integral to both systemic and localized immunity, safeguarding against infections and chronic inflammatory conditions (Table 2). While Table 1 summarizes the major gut microbial taxa and their immunological roles, Table 2 focuses on the functional and systemic immunological consequences of gut microbiome alterations that are relevant to TB susceptibility.

**TABLE 2:** Functional and systemic immunological consequences of gut microbiome alterations relevant to tuberculosis.

Immune response	Impact on the immune system	Mechanism	Reference
Innate immunity	Prevents systemic inflammation by strengthening intestinal barrier integrity	SCFAs, produced by the gut microbiome, enhance gut barrier integrity by promoting the expression of tight junction proteins. These proteins are crucial for maintaining the tight junctions between epithelial cells, which form a barrier that prevents the translocation of harmful substances, such as LPS, into the bloodstream	[44, 62, 64, 65]
	Modulates cytokine levels, reducing chronic inflammation risk	A healthy gut microbiome maintains a balance between proinflammatory cytokines, such as IL‐6 and TNF‐α, and anti‐inflammatory cytokines, like IL‐10	[52, 70–72]
	Promotes immune homeostasis and prevents overgrowth of pathogenic bacteria	The gut microbiome produces antimicrobial peptides that regulate microbiome composition. Moreover, beneficial microbes compete with pathogens for nutrients and attachment sites on the intestinal epithelium, thereby inhibiting their growth and establishment	[52, 66]
	Reduces systemic inflammation, lowering the risk of chronic diseases	The gut microbiome produces SCFAs, particularly butyrate, which exert anti‐inflammatory effects by inhibiting the production of proinflammatory cytokines and enhancing the integrity of the intestinal barrier. It also reduces endotoxin translocation (e.g., LPS) by maintaining gut integrity	[11, 44, 61, 62, 65]

Adaptive immunity	Supports immune cell differentiation and immune tolerance through Tregs	The gut microbiome influences Treg differentiation, by producing SCFAs, particularly butyrate, that enhance the differentiation of Tregs from naive T cells in the gut. Tregs help to suppress inflammatory responses and promote tolerance to self‐antigens and commensal microbes, thereby preventing autoimmune reactions	[51, 73–75]
	Enhances mucosal immunity and immune tolerance via IgA production	The gut microbiome enhances IgA production through the interactions between gut microbiota and GALT. The presence of specific bacterial species stimulates the differentiation of B cells into IgA‐producing plasma cells. Secretory IgA binds pathogens, preventing their interaction with the intestinal epithelium, thereby neutralizing potential threats	[55–59]
		The presence of certain commensal bacteria has been linked to increased TGF‐β levels which are known to enhance IgA class switching in B cells	

### 3.3. The Gut Microbiome and Its Role in Modulating Immunity Against TB

The gut microbiome plays an important role in host defense against infections, including TB, by modulating the immune response, producing antimicrobial compounds, and maintaining gut barrier integrity (Table 2). Certain gut bacteria enhance IFN‐γ production, activating macrophages to eliminate intracellular pathogens, including *Mtb* [76, 77]. In experimental settings, specific gut microbiota have also been associated with the development of lung‐resident memory T cells, which may contribute to longer‐term protection against TB.

Recent research has increasingly highlighted the role of the gut microbiome in modulating host immunity, particularly in the context of infectious diseases such as TB. The interplay between gut microbial communities and the host immune system is now recognized as a critical factor influencing both susceptibility to *Mtb* infection and the outcome of TB treatment. Through mechanisms such as microbial metabolites and immune regulation, the gut microbiome may contribute to both protective immune responses and, under certain conditions, disease progression.

One key mechanism by which the gut microbiome may influence host defenses against TB is by producing SCFAs, such as butyrate and propionate. These metabolites, generated during the fermentation of dietary fibers by gut bacteria, can modulate immune function by promoting Treg development, modulating effector T‐cell responses, and limiting excessive inflammation [48, 78]. The relevance of SCFAs to TB immunity is supported by findings from Maji et al., who reported that reduced abundance of SCFA‐producing bacteria was associated with compromised immune responses in individuals exposed to *Mtb* [79]. Similarly, Mori et al. observed that SCFAs can shape adaptive immune responses in a context‐dependent manner that may, under certain conditions, inadvertently support *Mtb* persistence [80].

Further supporting the immune‐regulatory role of gut microbiota, Yang et al. demonstrated that the microbiome can influence protective immune response against TB through the modulation of long noncoding RNAs, suggesting that microbial metabolites affect not only immune cells but also gene regulatory networks [81]. Negi et al. reported that perturbations of the gut microbiota were associated with impaired clearance of *Mtb* in experimental models, linking microbiome integrity to effective immune responses [82]. In line with these observations, Hagan et al. described how SCFAs may enhance the activity of both Tregs and effector T cells, which are involved in controlling *Mtb* infection and limiting disease progression [83].

Beyond their localized effects in the gut, microbial metabolites may influence distant organs, including the lungs, via the gut–lung axis. This bidirectional communication pathway enables gut‐derived signals to modulate pulmonary immune responses. He et al. reported that specific beneficial gut bacterial populations were reduced in TB patients, suggesting metabolic alterations that may be associated with impaired lung immunity [39].

#### 3.3.1. SCFAs in TB Immunity

SCFAs, mainly acetate, propionate, and butyrate, are metabolites produced by gut microbiota through the fermentation of dietary fiber and play context‐dependent roles in immune regulation during *Mtb* infection. Depending on the immunological and microbial context, SCFAs can either support or suppress immune responses.

##### 3.3.1.1. Enhancing Immunity

SCFAs have been shown to support immune defense in certain contexts by stimulating Th1 responses, which are essential for *Mtb* control. They can activate G protein‐coupled receptors (GPR41/43), leading to increased IFN‐γ production and enhanced macrophage activation [33, 84]. SCFAs have also been reported to support macrophage polarization toward an M1 phenotype [20] and to influence naive T‐cell differentiation toward Th1 and CD8+ T‐cell lineages, thereby strengthening cell‐mediated immunity [85, 86].

##### 3.3.1.2. Suppressing Immunity

Conversely, elevated SCFA levels can induce Tregs, which suppress inflammation but may also weaken anti‐*Mtb* responses [29, 87, 88]. In coinfections such as helminthiasis, SCFAs may further skew immunity toward a Th2‐dominant profile, increasing IL‐10 production and dampening Th1 responses and macrophage activity against *Mtb* [89, 90]. These findings highlight the complex and context‐dependent nature of SCFA‐mediated immune modulation. Their immunological effects are shaped by factors such as SCFA concentration, coinfection status, and overall immune balance, suggesting that therapeutic modulation of SCFAs would require careful contextual consideration to enhance TB immunity while minimizing potential suppressive effects.

#### 3.3.2. Alterations in the Gut Microbiome due to Helminth Infection

Helminth infections have a substantial impact on the gut microbiome, exerting both beneficial and detrimental influences on the composition and function of gut microbial communities. These parasites, including roundworms, hookworms, and flatworms, can modulate the host immune system in ways that may either support microbial balance or contribute to dysbiosis, depending on factors such as helminth species, infection intensity, and host immune responses.

#### 3.3.3. Beneficial Impact of Helminth Infection on the Gut Microbiome

Helminth infections can influence gut microbiome composition and diversity, in some contexts, enhancing beneficial bacterial populations while reducing the abundance of potentially harmful taxa. This modulation depends on helminth species, host immune response, and environmental conditions, and several studies have reported increased microbial diversity during helminth infection [9, 91] (Table 3).

**TABLE 3:** Beneficial impact of helminth infection on the gut microbiome.

Helminth impact on microbiome	Mechanism	References
Enhanced microbial diversity	Helminth infections generally increase microbial diversity, fostering a balanced gut ecosystem	[9, 91]
SCFA production	Helminths enhance SCFA production either directly or by promoting bacteria that produce these metabolites, thereby supporting gut health, immune tolerance, and anti‐inflammatory responses	[11, 64, 66]
Gut permeability and mucosal integrity	Helminths stimulate mucin production, protecting the gut lining and fostering beneficial bacteria, such as *Akkermansia*, that support gut barrier function and metabolic health	[92–94]
Interaction with dietary components	Helminths interact with diet to enhance SCFA production, demonstrating the role of diet in modulating microbiome responses	[95–97]

Helminths modulate the immune system, often inducing a Th2‐dominant response characterized by increased IL‐4 and IL‐10 production [98]. This Th2‐skewed environment has been associated with changes in gut microbial composition, including increased abundance of taxa such as *Lactobacillus* and *Bacteroidetes* that are linked to immune regulation [9, 99]. Such immunologically mediated shifts may contribute to a more regulated microbial environment under certain conditions. Helminths may also influence microbial composition by altering SCFA production, either directly or indirectly by promoting microbial populations capable of producing these metabolites. SCFAs are involved in gut barrier maintenance, immune tolerance, and regulation of inflammatory responses [11, 64, 66]. Helminth‐associated changes in SCFA levels may contribute to immunoregulatory effects within the gut, although outcomes appear to be context dependent [100].

Helminths may further influence the gut microbiome by modifying gut permeability and mucosal integrity. Helminth infections have been reported to stimulate mucin production, which may support the expansion of mucin‐utilizing bacteria such as *Akkermansia*, a genus associated with gut barrier maintenance and metabolic regulation [93, 94]. These changes may alter mucosal–microbial interactions, though their functional implications vary across studies.

The impact of helminths on microbiome composition varies according to helminth species and host immune response. In settings characterized by pronounced Th2 polarization, helminth infection has been associated with increased abundance of certain bacterial taxa, whereas altered or weakened immune responses may attenuate these effects, underscoring the need for context‐specific investigation [101].

Additionally, helminths may interact with dietary components in the gut, influencing the microbiome’s response to dietary fibers and complex carbohydrates. Such interactions can affect SCFA production, suggesting that diet may modulate helminth‐associated microbiome changes. However, evidence supporting dietary or microbiome‐targeted interventions in helminth infection remains preliminary [95–97].

Taken together, helminth infections can alter microbiome composition and diversity through immune modulation, metabolite production, and mucosal changes. These alterations may be associated with increased microbial diversity and shifts toward immunoregulatory taxa in certain contexts, although the effects vary across host populations and infection settings. Understanding these dynamics is important for interpreting helminth–microbiome interactions in endemic regions [9, 102, 103].

#### 3.3.4. Detrimental Impact of Helminth Infection on the Gut Microbiome

On the other hand, helminth infections can also have detrimental effects on the gut microbiome, particularly when they become chronic or are accompanied by excessive immune dysregulation (Table 4).

**TABLE 4:** Detrimental impact of helminth infection on the gut microbiome.

Helminth impact on microbiome	Mechanism	References
Altered gut microbiota composition	Helminth infections, such as those caused by *Schistosoma mansoni* and *Trichuris muris*, have been associated with reduced diversity and shifts in bacterial populations, favoring opportunistic pathogens like *Escherichia coli*	[10, 104]
Immune modulation causing dysbiosis	Helminths elicit a Th2‐skewed immune response characterized by increased IL‐4 and IL‐10 production, which may suppress Th1‐associated pathways and be associated with shifts in microbial populations favoring opportunistic taxa over beneficial bacteria	[105]
Intestinal inflammation	Helminths have been reported to induce inflammation of the gut lining (e.g., egg deposition), which may disrupt epithelial barrier integrity and contribute to dysbiosis	[106, 107]
Increased susceptibility to infections	Dysbiosis leads to a weakened gut barrier, increasing bacterial translocation and susceptibility to systemic infections or exacerbating existing conditions such as IBD	[10, 107]
Disruption of gut homeostasis	Dysbiosis has been associated with metabolic disturbances that may contribute to chronic inflammatory conditions or metabolic disorders	[108, 109]

### 3.4. Impact on Gut Microbiota Composition

Several studies, including those involving *Schistosoma mansoni* and *Trichuris muris*, indicate that helminth infections can be associated with quantitative and qualitative changes in the gut microbiota [104]. For example, *S*. *mansoni* infection has been reported to correlate with altered microbiota composition, including reduced microbial diversity and shifts in specific bacterial taxa, such as increased *Escherichia coli* and reduced *Lactobacillus* [104].

#### 3.4.1. Mechanisms of Dysbiosis Induction

Dysbiosis is characterized by a loss of beneficial microbial species and an overrepresentation of pathogenic or opportunistic microorganisms.

Helminth infections can induce dysbiosis through several mechanisms, including

• Immune modulation: helminths elicit an immune response characterized by enhanced Th2‐type polarization. This Th2‐skewed response may suppress Th1‐associated pathways involved in pathogen control and has been associated with shifts in microbial populations, potentially favoring opportunistic taxa over beneficial bacteria. Such immune modulation is accompanied by increased secretion of cytokines such as IL‐4 and IL‐10, which can further influence gut microbial populations [105].
• Intestinal inflammation: certain helminths can trigger inflammatory responses that disrupt barrier integrity. For instance, egg deposition by some helminths has been associated with inflammation of the intestinal mucosa, which may contribute to dysbiosis [106, 107]. These inflammatory processes may increase intestinal permeability, permitting bacterial translocation and heightened susceptibility to secondary infections.

#### 3.4.2. Consequences of Dysbiosis

The resulting dysbiosis may have several health implications, including

• Increased susceptibility to infections: dysbiosis may enhance gastrointestinal permeability, allowing bacterial translocation that can contribute to systemic infections or exacerbate existing inflammatory conditions [10, 107]. For instance, helminth‐associated dysbiosis has been linked to compromised gut barrier integrity and increased susceptibility to opportunistic infections [10, 107].
• Altered immune responses: dysbiosis can further influence host immune regulation, potentially reducing the effectiveness of immune responses against infections and complicating immunological control during co‐occurring diseases, which may also affect treatment responses [10].
• Disruption of gut homeostasis: shift in gut microbial equilibrium may lead to unfavorable metabolic consequences for both the host and resident microbiota, potentially contributing to chronic inflammatory conditions that have been associated with dysbiotic states [108, 109].

Finally, while helminths may reduce inflammation in certain gut‐related autoimmune conditions, their impact on microbial diversity and gut function in the presence of coinfections or pre‐existing gastrointestinal disorders may lead to adverse outcomes. In such contexts, a shift towards an imbalanced microbiome may hinder recovery or exacerbate clinical symptoms, particularly in immunocompromised hosts.

## 4. Interaction Between Helminth Infection, Gut Microbiome, and TB Susceptibility

### 4.1. Helminth–Gut Microbiome Axis in TB Infection

Helminth infections may influence TB susceptibility and immune responses by altering gut microbiome composition and modulating systemic immunity. Typically, helminth infections are associated with a Th2‐biased immune response, characterized by increased Tregs and enhanced production of anti‐inflammatory cytokines such as IL‐10 [98]. Such immunological shifts may attenuate Th1‐associated responses that are important for TB control, potentially influencing the host’s ability to respond effectively to *Mtb* [99].

One proposed pathway involves helminth‐associated changes in the abundance of SCFA‐producing gut bacteria. SCFAs such as butyrate can enhance Treg differentiation and function, contributing to an anti‐inflammatory environment that may favor helminth persistence while attenuating proinflammatory Th1 responses involved in TB control [9, 110]. Elevated Treg activity has been associated with reduced macrophage activation and IFN‐γ production, both of which are important for TB containment [79]. Collectively, these immunological changes may create conditions less favorable to optimal *Mtb* clearance, although the available evidence remains largely associated.

Moreover, helminth infections have been reported to affect gut barrier integrity, potentially allowing microbial products such as LPS to enter the systemic circulation responses [111]. Such microbial translocation may contribute to systemic inflammation signaling, which in the context of TB could influence pulmonary immune responses [112]. Helminth‐associated alterations in the microbiome may also affect immune cell signaling pathways, further complicating the regulation of host responses to *Mtb*.

Helminth infections are frequently associated with gut microbial diversity, which may have mixed implications for TB susceptibility. While greater diversity is often linked to immune resilience, certain helminth‐altered bacterial profiles, such as increased abundance of *Prevotellaceae*, have been associated with immune responses that may be less effective for TB control [104, 113]. Shifts in microbiota composition can also influence nutrient metabolism, including the synthesis and availability of micronutrients essential for immune function. For example, alterations in gut bacteria may affect the vitamin availability required for macrophage activity, potentially influencing host responses to TB [91, 111].

This interplay between helminth‐driven immune modulation and changes in gut microbiome creates a complex immunological environment that may influence TB susceptibility and disease progression. Individuals with helminth–TB coinfections have been reported to experience poorer clinical outcomes in some settings, potentially related to Th2‐skewed immune responses that attenuate protective Th1 pathways [9, 114]. Additionally, alterations in the gut microbiome may influence host responses to TB treatments, although evidence regarding drug–microbiome interactions remains limited.

In summary, helminth infections can alter gut microbiome composition and systemic immune regulation, potentially influencing pulmonary immune responses and TB susceptibility. Th2 polarization, SCFA‐associated immunomodulation, changes in gut barrier integrity, and altered nutrient availability may collectively shape host–*Mtb* interactions. Recognizing the role of the helminth–gut microbiome axis in TB may inform integrated management approaches in endemic regions, although further mechanistic and clinical studies are required [115, 116].

### 4.2. Gut–Lung Axis and Pulmonary Immune Regulation

The gut–lung axis represents a bidirectional communication network through which alterations in gut microbiota composition and microbial metabolites influence pulmonary immune responses and respiratory disease outcomes. Increasing evidence indicates that immune cell trafficking, microbial‐derived metabolites, and systemic inflammatory mediators link intestinal microbial communities to lung immunity, shaping host susceptibility to respiratory infections [4, 11, 117, 118]. These interactions may modulate key immune pathways involved in host defense against *Mtb*, including Th1‐mediated responses and macrophage activation.

#### 4.2.1. Immune Cell Trafficking

**FIGURE 1:**
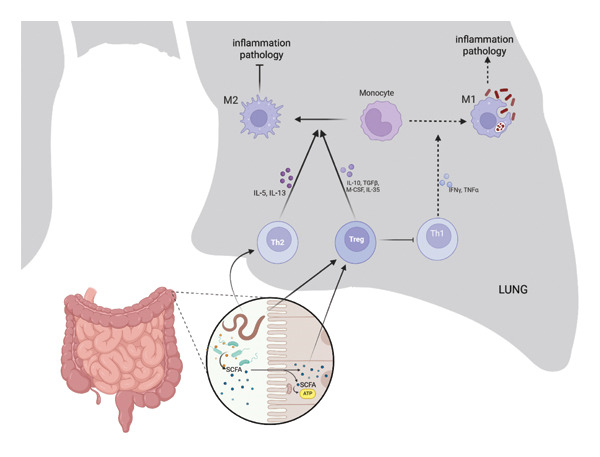
Proposed integrative framework illustrating how helminth infection may influence pulmonary tuberculosis susceptibility through modulation of the gut microbiome and immune regulation along the gut–lung axis. Helminth‐induced Th2 polarization and increased regulatory T‐cell (Treg) activity, partly mediated by microbiome‐derived short‐chain fatty acids (SCFAs), may attenuate Th1 responses essential for effective control of *Mycobacterium tuberculosis*. In addition, gut microbiome alterations may indirectly affect pulmonary immunity through systemic immune signaling and potential interactions with the lung microbial environment. This figure was created in BioRender. Berbudi [119] https://BioRender.com/e74l598.

One mechanism through which the gut may influence lung immunity is immune cell migration. Tregs and Th cells activated in the gut can migrate to the lungs, where they may influence local immune dynamics. Chemokine signaling guides this process, facilitating immune cell trafficking to pulmonary tissues. In TB, gut microbiota‐associated Treg activation has been proposed to contribute to increased Treg presence in the lung, which may attenuate Th1 responses important for *Mtb* control [117] (Figure 1).

#### 4.2.2. Microbial Metabolites and Lung Immunity

Microbial metabolites, particularly SCFAs, play an important role in gut–lung interactions and may contribute to host immune regulation during infections such as TB. SCFAs, including butyrate, produced by dietary fiber fermentation in the gut microbiota, can enter systemic circulation and influence immune responses in the lungs, including promoting Treg differentiation and modulating cytokine production. These immunomodulatory effects contribute to lung immune homeostasis and have been implicated in the regulation of inflammatory respiratory conditions [119]. However, elevated SCFA concentrations have also been associated with enhanced Treg induction and maintenance, which may suppress proinflammatory responses required for effective control of infections such as TB.

#### 4.2.3. Crosstalk and Bidirectional Regulation

The gut–lung axis functions bidirectionally, with lung infections or inflammation also influencing the gut microbiome. For instance, pulmonary inflammation has been associated with increased gut permeability, facilitating the translocation of microbial products such as LPS and contributing to systemic inflammation [120]. This process may promote gut dysbiosis, which can further modulate systemic inflammation and potentially affect both gut and lung immune environments [121]. Such inflammatory mediators may reach the lungs and influence immune responses during TB infection, although their contribution to tissue damage and disease progression appears to be context dependent.

#### 4.2.4. Impact on Respiratory Diseases

The gut–lung axis has been implicated in various respiratory diseases, including TB. Gut dysbiosis has been associated with altered lung immune responses, and specific gut microbial profiles may influence immunological pathways relevant to TB control [82, 122]. In TB‐endemic settings, variations in gut microbiota composition may contribute to interindividual differences in susceptibility and disease progression, particularly in regions where helminth infections further complicate gut–lung immune interactions.

#### 4.2.5. Gut–Lung Axis in Helminth–TB Coinfections

Helminth infections may further complicate gut–lung immune interactions in TB‐endemic areas by promoting Th2‐skewed responses that can attenuate Th1‐mediated immunity, which is essential for TB control. Helminths‐associated alterations in gut microbiota composition may enhance SCFA production, which has been linked to suppression of proinflammatory lung immune responses under certain conditions [123]. Additionally, helminth infections have been reported to reduce chemokines involved in Th1‐cell recruitment to the lungs, potentially impairing immune responses to *Mtb* [124].

#### 4.2.6. Gut Barrier Integrity and Systemic Inflammation

Helminth infections have been reported to affect gut barrier integrity, potentially enabling systemic dissemination of microbial products such as LPS, which may influence lung immune responses during TB [125]. Helminth‐associated dysbiosis may attenuate Th1 responses required for TB control while also contributing to proinflammatory signaling that can complicate disease management [5, 126], underscoring the role of gut integrity in modulating lung immunity and TB susceptibility.

Taken together, the disruption of the gut–lung axis by helminth infections may influence TB susceptibility, although the extent and clinical relevance of these effects remain to be fully elucidated. Targeting the gut microbiome as part of therapeutic strategies may hold potential, but supporting clinical evidence is currently limited [120, 127].

### 4.3. Potential Pathways Linking Helminth Infection, Gut Microbiome, and TB Susceptibility

The link between helminth infections, the gut microbiome, and TB susceptibility involves complex pathways, including immune modulation, microbial metabolite production, systemic inflammation, and gut–lung crosstalk. Helminths can influence gut microbiota and host immune regulation, potentially creating immunological conditions that may affect susceptibility to *Mtb* [98, 128].

#### 4.3.1. Immune Modulation

Helminths are associated with a shift in immune responses toward a Th2‐skewed profile, characterized by increased Tregs and IL‐10 production, which may attenuate Th1‐associated responses important for TB control [5, 126, 128]. Helminth‐associated Treg expansion has been linked to reduced IFN‐γ production and diminished macrophage activation, potentially influencing *Mtb* containment [129, 130] (Figure 1). Collectively, these immunological shifts may create conditions that are less favorable for optimal Th1‐mediated immune responses against *Mtb*.

#### 4.3.2. Microbial Metabolites

As discussed in the previous section, SCFAs play a central role in the immune‐regulatory functions of the gut microbiota, including enhancing Treg activity and promoting anti‐inflammatory responses. However, excessive SCFA levels have been associated with inhibition of Th1 responses required for TB control [74, 87]. Helminth‐associated increases in SCFA‐producing bacteria may therefore influence immune responses, potentially suppressing proinflammatory lung immunity under certain conditions while maintaining gut homeostasis [12, 131] (Figure 1).

#### 4.3.3. Dysbiosis and Systemic Inflammation

Helminth infections have been associated with dysbiosis, which increases gut permeability and facilitates the translocation of microbial products such as LPS, contributing to systemic inflammatory signaling [28, 132, 133]. Chronic inflammation linked to dysbiosis may contribute to immune dysregulation or exhaustion, potentially complicating immune control of TB [98, 128].

#### 4.3.4. Gut–Lung Crosstalk

Helminth‐associated alteration in gut microbiota composition may influence immune cell migration and cytokine profiles in the lungs [41]. Changes in gut microbial communities have been linked to altered chemokine expression, which may affect Th1‐cell recruitment and pulmonary immune responses relevant to TB control [134].

#### 4.3.5. Helminth Larvae–Lung Microbiota Crosstalk

The lung microbiota, a diverse community of bacteria, fungi, and viruses, plays an important role in respiratory homeostasis and disease susceptibility, including TB. In healthy individuals, lung microbial communities are maintained through a dynamic process of microbial immigration and clearance balance through continuous migration and clearance, primarily originating from the upper respiratory tract and inhaled air [135–137]. Commonly reported dominant bacterial genera include *Streptococcus, Prevotella, Veillonella,* and *Haemophilus*.

Disruption of lung microbial balance (dysbiosis) has been associated with altered pulmonary immune responses, including inflammatory signaling and impaired pathogen clearance. In TB patients, shifts in lung microbiota composition, such as increased abundance of certain Firmicutes, have been reported and correlated with disease severity or treatment outcomes, although causal relationships remain unclear [138–140].

Beyond bacterial communities, some intestinal helminths, such as *Ascaris lumbricoides*, *Ancylostoma duodenale*, and *Strongyloides stercoralis,* undergo larval migration through the lungs during their life cycles. During this transient pulmonary phase, helminth larvae may induce localized immune responses, including Th2‐skewed inflammation characterized by cytokines such as IL‐4 and IL‐5 [9]. These immune alterations could influence the pulmonary microenvironment and, indirectly, interactions with resident microbial communities, although direct evidence linking helminth larval migration to lung microbiota dysbiosis remains limited.

Such immune‐mediated changes may influence pulmonary immune regulation and could potentially affect susceptibility to respiratory infections, including TB. However, current evidence supporting a direct role of helminth larvae in reshaping the lung microbiota or determining TB outcomes is largely indirect, derived from experimental or observational studies.

The role of microbiota in modulating immunity is further illustrated by mucosal‐associated invariant T (MAIT) cells, which participate in early immune responses against bacterial pathogens, including *Mtb* [140]. Disruption of gut microbial homeostasis has been associated with altered MAIT cell function and reduced immune efficacy in TB models, highlighting the interconnectedness of gut and lung microbial‐immune networks [134]. Gut‐derived microbial metabolites, including SCFAs, may further influence pulmonary immune activation or suppression relevant to *Mtb* control [141].

## 5. Implications for TB Control and Management

### 5.1. Challenges in Clinical Management

Helminth coinfections may complicate clinical management by influencing TB progression and symptomatology. Coinfected patients have been reported to present with atypical clinical features, which may contribute to delays in diagnosis and initiation of treatment. In some settings, helminth–TB coinfection has been associated with more severe disease manifestations, including extensive pulmonary involvement requiring intensified clinical management [30, 142]. These observations highlight the potential need for coordinated clinical approaches that consider both infections.

### 5.2. Implications for TB Control in Helminth‐Endemic Regions

In regions with a high burden of both infections, dual‐focused TB management strategies may warrant consideration. Incorporation of helminth screening alongside TB diagnostic and treatment programs has been proposed as a means to better characterize coinfected populations. Helminth‐associated immunomodulation may influence TB diagnostic performance and treatment responses, although available evidence is largely observational [35, 143]. Integrating helminth control measures into existing health programs may therefore offer opportunities to refine TB control strategies in endemic settings, pending further evaluation.

### 5.3. Integrating Helminth Control into TB Prevention Programs

Integrating helminth control into TB prevention programs have been proposed as a complementary strategy in regions where both infections are endemic. Helminth infections have been associated with immune modulation that may influence TB susceptibility and treatment responses, suggesting that addressing helminth burden could potentially support TB control efforts [30, 86]. However, the impact of integrated interventions on TB incidence and clinical outcomes remains to be established through well‐designed clinical and implementation studies.

#### 5.3.1. Immunomodulatory Effects of Helminths and TB Susceptibility

Helminth infections are associated with Th2‐skewed immune responses that may attenuate Th1‐mediated immunity, including IFN‐γ production, which is important for TB control [86, 129]. Reduction of helminth burden has been reported to partially restore Th1‐associated immune activity in some contexts, which may influence host responses to *Mtb*. Nevertheless, evidence linking helminth eradication to reduced TB susceptibility remains limited and context dependent [35].

#### 5.3.2. Improved TB Treatment Outcomes Through Helminth Eradication

Helminth coinfections are linked to poorer TB outcomes, including prolonged treatment duration and higher relapse rates in some populations [25]. Reduction of helminth burden through deworming has been proposed as a strategy that may alleviate helminth‐associated immunomodulation, potentially influencing host immune responses during TB treatment [144]. Deworming has also been associated with reduced inflammatory burden in certain settings, which may support pulmonary recovery during TB management, although direct evidence of improved TB outcomes remains limited [145–147].

#### 5.3.3. Epidemiological Evidence Supporting Helminth Control for TB Prevention

Epidemiological evidence suggests that helminth infections may be associated with an increased risk of TB progression from latent to active disease. Incorporation of helminth measures into TB prevention strategies has therefore been proposed as a potential approach to interrupt this pathway, although causal relationships have not been firmly established [30, 130]. While deworming campaigns have demonstrated effectiveness in reducing helminth prevalence, their impact on TB incidence remains uncertain and warrants further investigation [148].

#### 5.3.4. Public Health Initiatives and the Feasibility of Mass Deworming

From a policy perspective, integrating helminth control into TB programs aligns with current WHO recommendations on deworming in endemic settings [3], although its impact on TB outcomes requires further clinical evaluation. Mass deworming programs have been shown to effectively reduce helminth prevalence, especially among vulnerable populations. The World Health Organization supports mass deworming as a cost‐effective public health intervention in high‐burden settings. In regions where TB and helminth infection overlap, coordinated implementation of deworming alongside TB prevention efforts has been proposed as a means to address shared risk factors, although evidence for direct impact on TB outcomes remains limited [149].

### 5.4. Therapeutic Potential of Modulating the Gut Microbiome

Modulation of the gut microbiome using probiotics, prebiotics, or related interventions has been proposed as a potential adjunctive strategy for TB management [150, 151]. The gut microbiome plays an important role in regulating immune responses relevant to *Mtb* control, and helminth‐associated Th2‐skewed immunity may influence this balance [142, 143]. Accordingly, microbiome‐targeted interventions may help modulate immune responses and inflammatory pathways, although their clinical utility in TB treatment remains to be validated.

#### 5.4.1. Immune Modulation Through Probiotics and Prebiotics

Certain probiotic strains, such *as Lacticaseibacillus rhamnosus,* have been reported to influence Th1‐associated cytokine responses in experimental settings, suggesting a potential role in supporting immune defenses against *Mtb* [152, 153]. Such effects may partially counterbalance Th2‐skewed immune responses associated with helminth infection, although clinical relevance remains to be determined. Prebiotics and nondigestible dietary fibers promote SCFA production, which can support Treg activity and anti‐inflammatory immune responses and may help modulate TB‐associated inflammation [154, 155].

#### 5.4.2. Restoring Gut Microbiome Balance

Gut dysbiosis has been reported in TB patients, particularly in the presence of helminth coinfection. Probiotics and prebiotics have been shown to promote the growth of beneficial bacterial taxa, such as *Bifidobacterium*, which may support gut barrier integrity and reduce systemic inflammatory signaling [156–158]. By modulating gut microbial composition, these interventions may influence systemic immune regulation, although their impact on TB recovery remains to be fully established.

#### 5.4.3. Reducing Systemic Inflammation

Reducing inflammation is an important consideration in TB management, as chronic inflammation has been associated with disease severity and delayed recovery. Probiotics can produce SCFAs that support anti‐inflammatory signaling pathways and may contribute to improved gut barrier integrity, potentially limiting the translocation of inflammatory endotoxins into the circulation [159]. Through these mechanisms, probiotics may influence host inflammatory responses during TB, although direct effects on *Mtb* control and treatment outcomes remain to be established.

#### 5.4.4. Enhancing Nutritional Status

Prebiotics have been reported to support nutrient absorption by promoting the growth of beneficial gut bacteria involved in carbohydrate metabolism and vitamin synthesis. Improved nutritional status is an important determinant of immune competence and has been associated with better clinical recovery in TB patients [160]. Accordingly, prebiotic interventions may help support gut health and nutritional status in helminth‐endemic settings, although their direct impact on TB treatment outcomes requires further evaluation.

#### 5.4.5. Potential for Combination Therapy and Host–Microbiota Directed Therapies

Host–microbiota directed therapy (HMDT), which combines microbiome‐targeted interventions with conventional TB treatments, has been proposed as a potential adjunctive approach. Certain probiotics have been reported to influence Th1‐associated immune pathways in experimental settings, suggesting a possible interaction with host responses during anti‐TB therapy [161, 162]. Other interventions, such as fecal microbiota transplantation (FMT), have been explored as a means of restoring microbial balance in patients with severe dysbiosis, but their application in TB remains experimental and requires careful evaluation [163, 164]. Microbiome‐focused therapies may offer supportive benefits through immune modulation, inflammation control, and nutritional support, although their clinical effectiveness in TB management has yet to be established.

### 5.5. Potential for Synergistic Benefits Beyond TB Control

Deworming has been associated with improvements in nutritional status through the reduction of parasite burden and may support overall immune function. Restoration of gut health following deworming has also been linked to changes in microbiome composition and inflammatory profiles, which could potentially influence susceptibility to infections, including TB [165].

However, the extent to which these effects translate into meaningful reductions in TB risk remains uncertain.

### 5.6. Research and Operational Considerations

Further research is needed to clarify the impact of deworming on TB‐related outcomes. Longitudinal and interventional studies may help define the effects of helminth control on TB incidence, disease progression, and treatment responses. In addition, operational challenges—including coordination of deworming activities with TB programs, resource allocation, and sustainable funding must be addressed to ensure feasibility and cost‐effectiveness [34].

Implementation of combined deworming and TB control strategies requires careful planning, context‐specific adaptation, and monitoring to optimize effectiveness. Public health education and community engagement may further support adherence to deworming initiatives. In conclusion, integrating helminth control into TB prevention and management programs represents a potentially complementary approach in coendemic regions. While such strategies may offer broader public health benefits, their impact on TB outcomes requires confirmation through rigorous clinical and implementation research.

## 6. Concluding Remarks

### 6.1. Summary of Key Findings

Helminth infections are associated with substantial modulation of the immune system by promoting a Th2‐skewed response, which may attenuate the Th1‐mediated immunity important for controlling TB. This immunomodulation has been linked to altered host immune responses to *Mtb* and, in some contexts, has been associated with more severe TB manifestation in coinfected individuals. Furthermore, helminth infections can alter the gut microbiome, potentially influencing TB susceptibility through immune modulation mediated by SCFA production. They can also be associated with microbial dysbiosis, disrupting immune balance and potentially contributing to TB disease progression.

### 6.2. Research Gaps and Future Directions

Future research should focus on elucidating the mechanisms by which helminth‐associated alterations in the gut microbiome influence TB pathogenesis, particularly within the framework of the gut–lung axis. Although numerous mechanistic insights have been derived from animal and murine models, important differences in immune architecture, microbiome composition, helminth burden, and disease progression limit the direct extrapolation of these findings to human TB. These translational challenges underscore the need for well‐designed human and translational studies to validate observations from experimental models.

The context‐dependent role of SCFAs in immune regulation, encompassing both immunostimulatory and immunosuppressive effects, warrants further investigation to clarify their relevance in TB. Additional studies are needed to assess how helminth coinfections affect TB outcomes across populations with differing immune statuses, including individuals living with HIV or suffering from malnutrition. Investigation of microbiome‐modulating interventions, such as probiotics and prebiotics, represents a potential avenue for future research, although their clinical utility in TB management remains to be established.

### 6.3. Clinical Implications

In regions where both helminth infections and TB are prevalent, healthcare providers may need to consider the potential influence of helminth coinfections on TB treatment and immune responses. Helminth‐associated immune modulation, particularly attenuation of Th1‐mediated pathways, may affect host responses to *Mtb*.

Management of helminth infections through deworming has been proposed as a strategy that may help partially restore immune balance in certain contexts, although evidence for direct improvements in TB outcomes remains limited.

In addition, modulation of the gut microbiome using probiotics or prebiotics has been suggested as a supportive approach to influence immune regulation and inflammatory responses in coinfected patients. While such interventions may offer potential benefits, their effectiveness in enhancing TB treatment outcomes requires validation through translational studies and well‐designed clinical studies.

### 6.4. Implications for Public Health and Policy

To address the dual burden of TB and helminth infections, public health may consider greater coordination between TB control and helminth control programs in coendemic settings. Mass deworming campaigns, when implemented alongside TB screening and treatment, have the potential to address shared risk factors and improve overall population health, although their direct impact on TB transmission and prevalence remains to be clarified. Policymakers may explore the feasibility of incorporating helminth control measures within broader TB control frameworks, particularly in high‐burden areas. Public health education and community engagement could further support adherence to integrated interventions, contributing to more comprehensive disease management.

These considerations are aligned with the Sustainable Development Goals (SDGs), particularly SDG 3 (Good Health and Well‐Being), including Target 3.3, which aims to end the epidemics of TB and neglected tropical diseases. In helminth–TB coendemic settings, integrated approaches may support disease control while also helping to reduce health inequalities.

## Funding

This study was funded by the Universitas Padjadjaran, 5317/UN6.C/PT.02/2025, and the Indonesian Endowment Fund for Education (LPDP), 4303/B3/DT.03.08/2025 and 3927/UN6. RKT/HK.07.00/2025.

## Conflicts of Interest

The authors declare no conflicts of interest.

## Data Availability

This article is a narrative review and does not report the generation or analysis of any new datasets. Therefore, data sharing is not applicable to this study.
